# Application of Mesenchymal Stem Cells in Inflammatory and Fibrotic Diseases

**DOI:** 10.3390/ijms21218366

**Published:** 2020-11-07

**Authors:** Jae-Sung Ryu, Eun-Jeong Jeong, Jong-Yeup Kim, Soon Ju Park, Won Seok Ju, Chang-Hyun Kim, Jang-Seong Kim, Young-Kug Choo

**Affiliations:** 1 Department of Otorhinolaryngology-Head and Neck Surgery, College of Medicine, Konyang University, Daejeon 35365, Korea; jsryu@kyuh.ac.kr (J.-S.R.); jykim@kyuh.ac.kr (J.-Y.K.); 2 Department of Biomedical Informatics, College of Medicine, Konyang University, Daejeon 35365, Korea; 3 Department of Biological Science, College of Natural Sciences, Wonkwang University, Iksan 54538, Korea; ej0314@kribb.re.kr (E.-J.J.); sjpark@wku.ac.kr (S.J.P.); jws7895@naver.com (W.S.J.); 4 Biotherapeutics Translational Research Center, Korea Research Institute of Bioscience and Biotechnology, Daejeon 34141, Korea; jangskim@kribb.re.kr; 5 Institute for Glycoscience, Wonkwang University, Iksan 54538, Korea; 6 College of Medicine, Dongguk University, Goyang 10326, Korea; ctlkim@dongguk.edu; 7 Department of Functional Genomics, University of Science and Technology (UST), Daejeon 34141, Korea

**Keywords:** mesenchymal stem cells (MSCs), paracrine factors, inflammatory disease, fibrotic disease

## Abstract

Mesenchymal stem cells (MSCs) are multipotent stem cells that can be isolated from various tissues in the adult body. MSCs should be characterized by three criteria for regenerative medicine. MSCs must (1) adhere to plastic surfaces, (2) express specific surface antigens, and (3) differentiate into mesodermal lineages, including chondrocytes, osteoblasts, and adipocytes, in vitro. Interestingly, MSCs have immunomodulatory features and secrete trophic factors and immune receptors that regulate the microenvironment in host tissue. These specific and unique therapeutic properties make MSCs ideal as therapeutic agents in vivo. Specifically, pre-clinical and clinical investigators generated inflammatory and fibrotic diseases models, and then transplantation of MSCs into diseases models for therapeutic effects investigation. In this review, we characterize MSCs from various tissues and describe their applications for treating various inflammation and fibrotic diseases.

## 1. Introduction

Stem cells are characterized by two specific traits: (1) ability to self-renew and (2) varied potency to differentiate into multilineage cells [1]. Based on their origin, stem cells can be grouped into three broad categories: embryonic stem cells (ESCs), induced pluripotent stem cells (iPSCs), and adult stem cells [2,3]. ESCs and iPSCs are pluripotent stem cells. ESCs are isolated from the inner cell mass (ICM) of blastocysts [4,5]. In contrast, iPSCs are produced from adult somatic cells that are genetically reprogrammed to an ESC-like state by ectopic expression of octamer-binding transcription factor 3/4 (OCT3/4), SRY-related high-mobility group box protein-2 (SOX2), oncoprotein c-MYC, and Kruppel-like factor 4 (KLF4) [6,7]. These stem cells can differentiate into cells of the three germ layers: ectoderm, mesoderm, and endoderm. Consequently, stem cells are considered of great interest for cell therapy and regenerative medicine. However, ESCs and iPSCs exhibit immunological rejection and genetic instability, respectively. In addition, therapeutic cell transplantation of ESCs or iPSCs leads to spontaneous teratomas and tumor development [8]. Specifically, several ethical concerns still shadow the use of ESCs [9]. However, transplantation of adult stem cells circumvents the immunological rejection, genetic instability, and teratoma formation, characteristic of ESCs and iPSCs. Therefore, many researchers have investigated adult stem cells owing to their biological importance and clinical applications.

In this review, we summarized the minimal criteria for cell therapy and potential applications of adult stem cells in inflammatory and fibrotic diseases using various animal models, focusing specifically on mesenchymal stem cells (MSCs).

## 2. Human Mesenchymal Stem Cells (hMSCs)

### 2.1. Criteria for the Characterization of hMSCs for Clinical Applications

**Figure 1:**
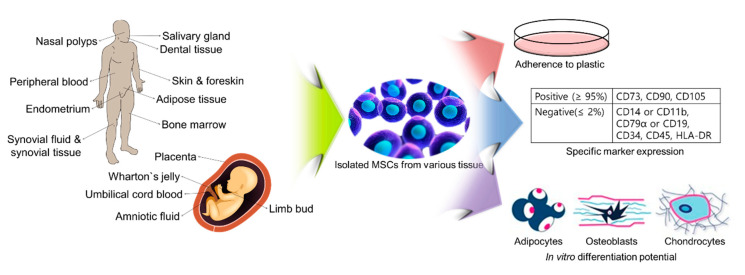
Characteristics and source of isolation of human mesenchymal stem cells (hMSCs).

MSCs are well-known adult stem cells that have self-renewal potential and the ability to differentiate into cells of mesodermal lineage, such as chondrocytes [10,11], osteoblasts [12,13,14], and adipocytes in vitro [15,16]. Specifically, there is a need to define minimum criteria for the use of hMSC in therapy, which was declared in 2006 by the International Society for Cellular Therapy (ISCT) [17]. Three criteria were defined for hMSCs: (1) adherence to tissue culture flask when maintained in standard culture conditions; (2) over 95% of the MSC population must express specific surface antigens (CD73, CD90, and CD105), but not CD14 or CD11, CD19 or CD79α, CD34, CD45, or human leukocyte antigen-DR (HLA-DR) (under 2% positive); and (3) MSCs must differentiate into mesodermal lineage cells, such as chondrocytes, osteoblasts, or adipocytes in vitro, under standard differentiation conditions (Figure 1 and Table 1).

### 2.2. Isolation of hMSCs from Various Tissues

Since the first description of hMSCs isolated from bone marrow [18,19,20,21,22,23,24,25,26,27,28,29,30,31,32,33,34], many pre-clinical and clinical researchers isolated and characterized MSCs from various tissues, such as umbilical cord blood [24,26,35,36,37,38,39,40,41,42,43,44], adipose tissue [24,26,45,46,47,48,49,50,51,52,53,54], Wharton’s jelly [55,56,57,58,59,60,61,62], amniotic fluid [63,64,65], dental tissue [12,13,66,67,68,69,70,71,72,73,74], skin and foreskin [75,76], placenta [36,77], salivary gland [78,79], synovial fluid [80,81], synovial tissue [10,11,82,83], endometrium [84,85], limb bud [86], peripheral blood [87,88,89,90], and nasal polyps [91,92,93,94] (Figure 1 and Table 1).

## 3. Mesenchymal Stem Cells and Inflammatory Diseases

Inflammation is a protective response to harmful external stimuli and aids tissue repair and remodeling, however, when dysregulated can have detrimental effects [95]. In fact, excessively prolonged dysregulation of the immune system can lead to a vast array of inflammatory and autoimmune disorders, such as graft-versus-host disease (GVHD), multiple sclerosis (MS), type 1 diabetes (T1D), joint diseases, inflammatory bowel diseases (IBD), systemic lupus erythematosus (SLE), and chronic rhinosinusitis with nasal polyps (CRSwNP) [95,96]. In this chapter, we summarize what is currently known about the therapeutic effectiveness of MSCs in animal models of several immune-mediated diseases (Table 2).

### 3.1. Graft-Versus-Host Disease (GVHD)

GVHD is a major complication that occurs after transplantation, and is the result of donor-derived immune cells mounting an alloreactive response against host tissues and organs [97]. GVHD animal models are generated by depleting endogenous hematopoietic cells by radiation or chemotherapy, followed by the reconstitution of the immune system based on allogeneic bone marrow transplantation [98].

Miyashima investigated the transplantation of MRL/lpr mouse bone marrow-derived MSCs into irradiated recipients, leading to a GVHD-like wasting disease, but irradiated recipient animals survived much longer when the bone marrow transfer was accompanied by a bone graft [99]. In an MHC-mismatched model, in which C3H/He mice-derived MSCs were transplanted into irradiated BALB/c mice, the infusion of bone marrow-derived MSCs into the bone marrow allowed recipient mice to survive much longer than those receiving only bone marrow cells [100]. These results showed that immortalized MSC lines could suppress GVHD in this model as well [101]. T cell-derived interferon-gamma (IFN-γ) is an important factor; MSCs treated with IFN-γ prior to infusion are superior to untreated MSCs for increasing survival after bone marrow transplantation [133]. When recipients received transplantation with IFNγ-deficient T cells, MSCs were unable to enhance survival. Moreover, treatment of MSCs with IFN-γ prior to infusion enhanced the immunosuppressive capacity of MSCs; thus, IFNγ-treated MSCs suppressed GVHD even when far fewer cells had been administrated [134]. It is suggested that activation-induced production of cytokines may be required for maximal immunosuppression of MSCs in vivo [133].

### 3.2. Multiple Sclerosis (MS)

MS is a central nervous system (CNS) disorder, characterized by progressive demyelination of the nerves from the spinal cord and brain [135]. Most frequently, the MS animal model is experimental autoimmune encephalomyelitis (EAE), a model of induced CNS inflammation [115,116].

Most studies have shown that MSC-based therapy in EAE has potent immunosuppressive effects. Bone marrow-derived MSCs transplanted to mice showed a significantly milder disease course than untreated animals in a progressive EAE model [117]. Moreover, specifically bone marrow- and adipose tissue-derived MSCs, effectively suppressed EAE in a relapsing and remitting model [118,136,137,138].

A reduction in the secretion of inflammatory cytokines by T cells accompanies a decrease in disease activity, and T cells in MSC-transplanted mice appear to be hyporesponsive to antigenic stimulation or anergic [117,118,139]. Murine MSCs were shown to mediate immune suppression, at least in part, by a novel pathway inhibiting chemokine (C–C motif) ligand 2 (CCL2; monocyte chemoattractant protein 1, MCP1) [139]. MSCs secrete several matrix metalloproteinases (MMPs), which can cleave MSC-derived CCL2. Consequently, this inhibits, rather than activates, C–C chemokine receptor type 2 (CCR2)-expressing immune cells [140]. Moreover, MSCs derived from ongoing sick donors are unable to suppress disease upon transplantation to autologous recipients; thus, a defect in MSC function may play an important role in the pathogenesis of EAE [117].

### 3.3. Type 1 Diabetes (T1D)

Diabetes mellitus is classified into two types: type 1 diabetes (T1D) and type 2 diabetes (T2D). Specifically, T1D is characterized by an immune-mediated response against insulin-producing pancreatic β-cells [102]. T1D animal models are generated by the iterative treatment of streptozotocin (STZ) and damaged β-cells. This damage attracts immune cells, which leads to insulitis, and eventually to immune-mediated β-cell destruction. In the STZ-induced mouse model, syngeneic bone marrow-derived MSCs reverted hyperglycemic animals to normal blood glucose levels [103]. Autologous bone marrow-derived MSC transplantation led to increased insulin secretion and sustained normoglycemia, with a shift in T cell cytokine production toward that of TH2 cells in an STZ-induced T1D rat model [107]. MSC transplantation homed to the pancreatic and kidney islets, promoted tissue repair, and increased insulin production and renal function in STZ-treated mice [103,108].

Transplantation of MSCs with islet allografts significantly enhanced the long-term survival of STZ-induced diabetes models in rats and mice [104,109]. In a non-human primate model, allogeneic bone marrow-derived MSC and intraportal islet transplantation significantly enhanced islet engraftment and function, which was associated with an increased number of regulatory T cells [105]. In addition, MSC transplantation led to a decrease in TH1-associated cytokines and an increase in interleukin 10 (IL-10)-producing regulatory T cells in rats [109]. However, mouse-derived MSCs mediate their immunosuppressive effects by the production of metalloproteinases that cleave the alpha chain of the IL-2 receptor (CD25) from the surface of activated T cells, thus leaving T cells hyporesponsive to IL-2 [104].

The non-obese diabetic (NOD) mouse strain is an animal model for spontaneous autoimmune diabetes, and this disease animal model appears to share many features of T1D in humans. Transplantation of MSCs to NOD mice has been shown to protect them before disease onset and even cure it when administered after the onset of hyperglycemia [106,141,142]. NOD mouse-derived MSCs were unable to suppress the disease in recipients, but BALB/c mouse- or NOD-resistant mouse-derived MSCs were able to suppress disease; thus, transplantation of MSCs into NOD mice may have a defect in their ability to suppress immune responses [141]. In addition, MSC treatment was associated with a reduction in the frequency of inflammatory CD4+ T cells and an increase in the frequency of regulatory T cells [106,141,142]. These results demonstrate that MSC-based therapy suppresses the autoimmune attack of endogenous β-cells and improves the maintenance of allogeneic islet allografts in T1D animal models.

### 3.4. Joint Diseases: Osteoarthritis (OA) and Rheumatoid Arthritis (RA)

OA is the most common joint disease that is generated by the gradual deterioration of the cartilage in joints. Specifically, OA is defined by cartilage degradation progression, subchondral bone remodeling, bone marrow lesions, meniscal damage and synovitis [143,144]. This disease subsequently induces an immune response with further damage to the joint [145]. However, MSCs are playing immunoregulatory and suppress all immune cells, thus MSCs transplantation inhibits OA progression and differentiates into chondrocytes. In cell-to-cell contact (juxtacrine) and production of trophic soluble factors (paracrine) manner, MSCs are inhibited production and migration of tumor necrosis factor-alpha (TNF-α), production and activation of IFN-γ, and activation and proliferation of B cells, however, activated production of IL-4 and IL-10, and proliferation of T regulatory (T reg) cells [144]. Furthermore, in paracrine mechanisms, MSCs may modulate the function of immune cells in a cell-to-cell contact-dependent manner [144,146]. Interestingly, MSCs have been demonstrated to promote tissue regeneration and immunosuppression in OA animal models, and clinical trials have been registered for OA in humans [110,111,112,147]. Several preclinical studies investigated that intra-articular injection of autologous MSCs from expanded in vitro effectively reduced cartilage degradation and joints inflammation in various animals [113,114]. Intra-articular transplanted MSCs successfully engraft in the injured site of cartilage and promote its regeneration and repair [148]. Specifically, MSCs transplantation into damaged intra-articular showed benefic effects, such as reduced cartilage degeneration, attenuated joint inflammation, improved clinical and radiographic symptoms and signs of OA [144].

Rheumatoid arthritis (RA) is a common autoimmune disease characterized by chronic joint inflammation. This disease is initiated when autoreactive T cells infiltrate the synovial tissue and secrete cytokines and chemokines into the joint [119]. However, several studies demonstrated that MSCs can regulate the immune system, and control inflammation. Thus, these mechanisms are inhibited function and proliferation of T and B cells, triggered the development of CD4+CD25+FoxP3+ T reg cells, and suppressed the maturation of dendritic cells [120,149]

An animal model of RA is the collagen-induced arthritis (CIA) model [121]. Intraperitoneal injection of allogeneic bone marrow-derived MSCs at the time of initial immunization significantly decreased the incidence of disease and showed a therapeutic effect in the CIA mouse model, such as diminished count of granulocyte-macrophage colony-stimulating factor-expression CD4+ T cells, the critical ells in the pathogenesis of RA [122,123,150]. In addition, transplantation of various doses of MSCs inhibited the signs of joint inflammation and the overall joints were mildly regenerated as compared with non-transplanted animals [124,125,151,152]. Furthermore, several studies have evidenced the immunomodulatory properties of MSCs in inflammatory arthritis via suppression of T-cell proliferation as well as the function of T reg cells [122,126,151]. Moreover, MSCs suppressed the potential of follicular helper T cells in contributing to B cells [127]. These evidence indicated that MSCs have the potential to control inflammation and might be helpful in ameliorating clinical symptoms of OA and RA patients.

### 3.5. Inflammatory Bowel Diseases (IBD)

IBD are characterized by destructive inflammation of the colon or small intestine in humans. An animal model of IBD can be generated by treatment with dextran sulfate sodium (DSS) added to drinking water, which causes chemical damage to the intestine, or by intrarectal administration of trinitrobenzene sulfonic acid (TNBS) [153]. MSC transplantation has suppressed most measurable disease outcomes and improved survival rate in IBD animal models, specifically, DSS-induced acute colitis models [154]. In addition, transplantation of MSCs has shown therapeutic effects following intrarectal administration of TNBS [155]. Interestingly, human bone marrow, gingiva, or umbilical cord blood-derived MSC transplantation were shown to suppress experimental colitis in a DSS-induced colitis mouse model [156,157,158,159].

Several studies have demonstrated that MSC infusion increased the frequency of regulatory T cells accompanied by a reduction in the number of T cells secreting inflammatory cytokines [154,155,160]. Fas ligand (FasL)-deficient mice-derived MSCs were not able to suppress disease in DSS-induced colitis models [159,160]. MSC transplantation in colitis models can induce FasL-mediated apoptosis in T cells, and this increase in the frequency of apoptotic cells indirectly leads to an increase in regulatory T cell number; thus, macrophages engulfing apoptotic T cells increase their production of TGFβ. Furthermore, the loss of Fas in MSCs disrupted the production of CCL2, suggesting that non-apoptotic Fas signaling is required for CCL2 secretion in MSCs. Therefore, Fas-deficient MSCs are incapable of attracting T cells in close proximity to FasL-mediated killing [160].

### 3.6. Systemic Lupus Erythematosus (SLE)

SLE is a complex autoimmune disease that causes progressive and profound damage to a variety of organs and tissues [161]. SLE animal models were generated by the progeny of a breeding pair consisting of a New Zealand Black (NZB) mouse and New Zealand White (NZW) mouse, or mutation in the gene encoding Fas (lpr) on the MRL strain background [162]. Hybrid mice (NZB/NZW F1) developed anti-nuclear and anti-DNA antibodies along with glomerulonephritis, as seen in patients with SLE. In MRL/lpr mice, the immune cells cannot undergo Fas-mediated apoptosis, favoring pronounced lymphoproliferative disorder that leads to the development of anti-nuclear antibodies and subsequent glomerulonephritis [162].

In the NZB/NZW F1 models, the results are ambiguous. However, human umbilical cord blood-derived MSCs had only a moderate effect on disease parameters and animal survival, despite markedly reducing serum levels of pro-inflammatory cytokines, such as IL-2, TNF-α, and IL-12, and increasing anti-inflammatory cytokine levels [163]. The infusion of allogeneic bone marrow-derived MSCs reduced serum levels of anti-DNA antibodies and improved the renal function in MRL/lpr models [164]. Human bone marrow or umbilical cord blood-derived MSCs suppressed disease and led to a reduction in anti-dsDNA antibodies, proteinuria, and renal pathology in the MRL/lpr models [165,166]. Furthermore, clinical improvement was accompanied by an increase in the frequency of regulatory T cells and a reduction in the number of IL-17 producing CD4+ T cells [164].

### 3.7. Chronic Rhinosinusitis with Nasal Polyp (CRSwNP)

Chronic rhinosinusitis (CRS) is one of the most common chronic inflammatory diseases of the sinonasal mucosa, and is characterized by an edematous mass of hyperplastic epithelium and lamina propria prolapse of the nose, leading to nasal obstruction, hypersecretion, loss of the sense of smell, and reduced quality of life [165]. CRS is a heterogeneous disease and is generally classified into two subtypes, CRS without nasal polyps (CRSsNP) and CRS with nasal polyps (CRSwNP), which have distinct inflammatory and remodeling profiles [166,167,168,169]. Moreover, CRSwNP can be further classified into two subtypes: eosinophilic CRSwNP (E-CRSwNP) and non-eosinophilic (or neutrophilic) CRSwNP (NE-CRSwNP) [170,171]. CRSwNP animal models were generated by administration of ovalbumin (OVA) and *Staphylococcus aureus* enterotoxin B (SEB) for E-CRSwNP or lipopolysaccharide (LPS) for NE-CRSwNP [128,129,130,131,132]. Nasal polyps (NPs) are unique abnormal lesions that grow from the lining of the nasal and paranasal sinuses by an innate response to exogenous proteases from allergens, such as pollen, mite, fungi, and microorganisms, and type 2 inflammation plays a critical role in NP development in patients [130]. Thus, NP tissues consist of various inflammatory cells, including B cells, natural killer (NK) cells, monocytes, dendritic cells, and Th lymphocytes. Specifically, type 2 cytokines, IL-4, IL-5 and IL-13, play important roles mediating inflammation in NP development, when inducing the epithelial-derived cytokines, such as IL-25, IL-33 and thymic stromal lymphopoietin (TSLP) that drive the activation of group 2 innate lymphoid cells (ILC2s) to release type 2 cytokines in an antigen-independent manner [32].

NP-derived cell culture with MSCs showed a significant decrease in the frequency of inflammatory cells and an increase in the frequency of Treg cells. Furthermore, MSCs inhibited the proliferation of CD4+ and CD8+ T cells and changed the global cytokine profile from a pro-inflammatory to an anti-inflammatory profile, as suggested by the increase in IL-10 and decrease in IL-2, TNF-α, and IFN-γ levels [49]. However, immune modulation of MSCs on CRSwNP are still unknown in pre-clinical and clinical studies.

## 4. Mesenchymal Stem Cells and Fibrotic Diseases

Fibrosis is characterized by excessive accumulation of extracellular matrix components and the development of fibrous connective tissue. Consequently, fibrosis induces disruption of tissue function in the affected organs, such as the lung, liver, pancreas, and heart. In this chapter, we summarize what is currently known about the therapeutic effectiveness of MSCs against fibrotic diseases (Table 3).

### 4.1. Lung Fibrosis

There is a number of lung fibrotic disease animal models for the five major pathologies defined, including bronchopulmonary dysplasia (BPD), acute respiratory distress syndrome (ARDS), chronic lower respiratory disease (CLRD), cystic fibrosis (CF), and idiopathic pulmonary fibrosis (IPF) [186,187,188,189,190]. To assess the therapeutic effect, MSCs have been transplanted into lung disease models via intravenous (IV), intratracheal (IT), intraperitoneal (IP), intranasal (IN) delivery, and bone marrow transplantation (BMT), and the following effects were observed: reduction of inflammation, fibrosis and pulmonary hypertension, an increase of survival rate and extracellular matrix production, protection of alveoli, and improved pulmonary functions [172,173,190,191,192,193]. The therapeutic effects of MSCs in lung disease have been demonstrated to act via a direct bystander paracrine mechanism and through differentiation of transplanted MSCs into the pulmonary epithelium. Several studies have shown that MSCs secrete various growth factors, such as hepatocyte growth factor (HGF), epithelial growth factor (EGF), keratinocyte growth factor (KGF), vascular endothelial growth factor (VEGF), insulin growth factor (IGF), angiopoietin-1, and adiponectin [174,175,176,177,178,179,194]. Moreover, occasional in vitro alveolar epithelium differentiated MSCs transplanted into an alveolar type-II phenotype with a minor contribution to epithelium repair [192,195].

### 4.2. Liver Fibrosis

Cirrhosis is the end stage of progressive fibrosis caused by nonalcoholic steatohepatitis (NASH), alcohol, and viral hepatitis. This disease will progress to hepatocyte loss and subsequent disruption of the hepatic vasculature. Liver transplantation is the most effective therapy for hepatic disease. However, this strategy is hindered by the lack of donor organs, high cost, and long-term treatment with immunosuppressants after transplantation. Thus, the therapeutic potential of MSCs has been investigated as well as their differentiation capacity, immunoregulatory properties, and secretion of trophic factors.

Several studies have demonstrated that MSCs are able to differentiate into hepatic cells and recover liver function by hepatic stellate cell apoptosis and decreasing the expression of transforming growth factor (TGF)-β and alpha-smooth muscle actin (α-SMA) [196,197,198]. Furthermore, hepatic differentiation of MSCs has been demonstrated in vivo, and various trophic and immunomodulatory factors play a key therapeutic role in the treatment of liver fibrosis. Trophic factors, including antiapoptotic factors, HGF and IGF, angiogenetic factor, VEGF, mitogenic factors, EGF, HGF, nerve growth factor (NGF), and TGF-α, are secreted from MSCs and prevent the apoptosis of hepatocytes [199,200]. Moreover, the transplantation of MSCs to patients with liver fibrosis showed clinical efficiency. The results seem to be a significant improvement in the model for end-stage liver disease (MELD) score and metabolic parameters [201,202,203,204,205].

### 4.3. Pancreatic Fibrosis

Pancreatic fibrosis is characterized by a constant histopathological feature of chronic pancreatitis of varying etiologies, and thus, many therapeutic studies have investigated the transplantation of MSCs for treating pancreatitis. Pancreatitis is characterized by the release of pancreatic digestive enzymes from damaged exocrine cells. Specifically, chronic pancreatitis leads to damage in both the endocrine and exocrine pancreatic tissues and can be triggered by risk factors, such as alcohol consumption, genetic mutations, and pancreatic duct obstruction.

A chronic pancreatitis animal model was generated by intravenous injection of dibutyltin dichloride via the penile vein in Sprague Dawley (SD) rats [180,181,206]. Transplantation of MSCs to chronic pancreatitis animal models showed reduced pancreatic damage and decreased fibrosis [180,181,206]. This effect was considered a result of the inhibition of pancreatic satellite cells. Moreover, transplanted MSCs engrafted damaged pancreatic tissue and lowered the expression of monocyte chemoattractant protein 1 (MCP-1) vascular cell adhesion molecule 1 (VCAM-1), IL-6, and TNF-α [181]. Nuclear factor kappa B (NF-κB), an important regulator of the inflammatory response and apoptosis, was inactivated in MSCs using the inhibitor IκBαM. When IκBαM gene-modified MSCs, IκBαM-MSCs, transplanted into animal models, reduced the levels of proinflammatory cytokines, such as IL-1, IL-6, IL-8, FN, TIMP-1, TIMP-2, TNF-α, CTGF, ICAM-1, and TGF-β1, but increased anti-inflammatory cytokines, such as IL-10, and promoted the apoptosis of pancreatic stellate cells [180].

### 4.4. Heart Fibrosis

Heart disease involves pathological myocardial remodeling characterized by excessive deposition of extracellular matrix proteins and cardiac fibrosis. Cardiac fibrosis is caused by multiple pathways, such as hormonal, mechanical, and inflammatory mechanisms [182]. Specifically, in the inflammatory response, fibroblasts proliferate in the heart and differentiate into myofibroblasts. Additionally, myofibroblast and cardiomyocyte interactions contribute to the adverse structural and functional abnormalities observed in heart disease, including aortic stenosis.

Many studies have shown that MSCs secrete various paracrine factors, such as HGF, VEGF, IL-6, migration-related chemokine stromal cell-derived factor (SDF)-1α, and brain-derived neurotrophic factor (BDNF), and modulate several key cell processes, such as protection and/or repair under different pathological conditions [183,184,185,207,208,209,210,211,212]. Interestingly, allogeneic MSC therapy improves the endothelial function in patients with heart disease since allogeneic MSCs secrete higher levels of nitric oxide and have reduced levels of circulating VEGF compared to autologous MSCs [208]. MSCs also stimulate the survival and proliferation of adult cardiomyocytes via Akt-mediated pathways, and consequently, MSCs promote endogenous cardiomyocyte regeneration [183,209,210]. Secreted SDF-1 from MSCs induces migration, proliferation, and cardiomyocyte differentiation [183,184,211]. These results indicated that persistent activation of SDF-1 with gene therapy may be less preferable than transient, cell-based approaches for the treatment of heart failure [212]. Moreover, MSCs can degrade the extracellular matrix and promote the reduction of fibrosis in scarred tissues [213]. Transplantation of MSCs to type I collagen present in fibrotic tissue upregulates dysregulation of myocyte regeneration and repair, but downregulates growth and inflammatory gene expression, resulting in decreased MSC-induced myoblast proliferation [214,215,216,217,218,219,220,221].

## 5. Conclusion

MSCs have been extensively used in regenerative medicine, as they are easy to isolate from various tissues and retain their ability to expand for long periods without losing their characteristics for applications in laboratory-based scientific and pre-clinical investigations. Moreover, these cells are able to differentiate into cells of the mesodermal lineage, secrete trophic factors related to immune regulation, and migrate toward sites of inflammation and/or damaged tissue. Therefore, MSCs have significant potential in regenerative medicine and more than 200 clinical trials aimed at treating a broad range of degenerative medicines [222]. This review summarized that many pre-clinical and clinical investigators focus on the production and secretion of immunomodulatory and cytoprotective trophic factors, thus they generated various animal models of inflammatory and fibrotic diseases, and then transplanted MSCs directly or indirectly into injured tissues. After MSCs transplantation, MSCs secreted various paracrine factors, and then provided protective microenvironmental effects, and accelerated the activation of local tissue-resident progenitor populations. These secreted paracrine factors from MSCs also provided protective microenvironmental effects and accelerated the activation of local tissue-resident progenitor populations. These properties indicate that MSCs will play an important role as therapeutic agents in vivo, especially for regenerating damaged or diseased cells. However, MSCs do not always show a positive role in various inflammatory and fibrotic diseases, because MSCs can induce tumorogenesis and immunogenesis in transplanted regions. Furthermore, MSCs can move other tissue from transplantation regions because they have homing properties. Therefore, the pre-clinical and clinical investigator must conduct tumorgenesis, immunogenesis, and distribution of transplanted MSCs in other tissues using humanized animals. Moreover, in order to use MSCs therapy, MSCs isolation and cultivation must be carried out at standardized good manufacturing practice (GMP) facilities, and cultivated MSCs must be tested for purity, potency, genetic stability and various microbial tests that include mycoplasma. For applications of these MSCs to humans, MSCs should be managed and used through applications and approval by the food and drug administration of each government.

## Figures and Tables

**Table 1:** Biological features of hMSCs from different sources, surface markers, and differentiation capacity.

Source	Cell Surface Markers		Lineage Differentiation	References
	Positive	Negative		
Bone marrow	SH2, SH3, CD29, CD44, CD49e, CD71, CD73, CD90, CD105, CD106, CD166, CD120a, CD124, STRO-1	CD14, CD34, CD45, CD19, CD3, CD31, CD11b, HLA-DR	Adipocytes, Chondrocytes, Osteoblasts, Hepatocyte, Cardiomyocytes, Pancreatic cells, Neuronal-like cells	[18,19,20,21,22,23,24,25,26,27,28,29,30,31,32,33,34]
Umbilical cord, Umbilical cord blood	CK8, CK18, CK19, CD10, CD13, CD29, CD44, CD73, CD90, CD105, CD106, HLA-I, HLA-II	CD14, CD31, CD33, CD34, CD45, CD38, CD79, CD133, vWF, HLA-DR	Adipocytes, Chondrocytes, Osteoblasts, Hepatocytes, Endothelial-like cells, Neuronal-like cells, Pancreatic cells	[6,24,35,36,37,38,39,40,41,42,43,44]
Wharton’s jelly	CD13, CD29, CD44, CD73, CD90, CD105, HLA-I	CD14, CD34, CD45, CD31, CD79, HLA-II, HLA-DR	Adipocytes, Osteoblasts, Chondrocytes, Hepatocytes, Neuronal-like cells	[55,56,57,58,59,60,61,62]
Adipose tissue	CD13, CD29, CD44, CD71, CD73, CD90, CD105, CD166, HLA-I, HLA-ABC, STRO-1	CD10, CD14, CD24, CD31, CD34, CD36, CD38, CD45, CD49, CD117, CD133, SSEA4, CD106, HLA-II, HLA-DR	Adipocytes, Chondrocytes, Osteoblasts, Hepatocyte, Cardiomyocytes, Pancreatic cells, Neuronal-like cells,	[24,26,45,46,47,48,49,50,51,52,53,54]
Amniotic fluid	SH2, SH3, SH4, CD, CD29, CD44, CD49, CD54, CD58, CD71, CD73, CD90, CD105, CD123, CD166, HLA-ABC, HLA-DR	CD10, CD11, CD14, CD31, CD34, CD49, CD50, CD117, HLA-DR, DP, DQ, EMA	Adipocytes, Osteoblasts, Neuronal-like cells	[63,64,65]
Dental tissues	CD29, CD44, CD90, CD105, SH2, SH3, CDHLA-DR, CD117, CD46, DPSC-EZ, DPSC-OG	CD10, CD14, CD34, CD45, HLA-DR, Stro-1, NGFR	Adipocytes, Chondrocytes, Osteoblasts, Pancreatic cells, Melanocytes, Neuronal-like cells	[12,13,66,67,68,69,70,71,72,73,74]
Skin and foreskin	CD44, CD90, CD73, CD105, CD166, SSEA4, Vimentin	CD14, CD45, CD34, c-kit, CD133, SSEA3, OCT-4, TRA 1–60, TRA 1–81, HLA-DR	Adipocytes, Osteoblasts, Chondrocytes, Myoblasts	[75,76]
Placenta	CD29, CD44, CD73, CD90, CD105	CD45, CD34, HLA-DR	Adipocytes, Osteoblasts, Endothelial-like cells, Neuronal-like cells	[36,77]
Salivary gland	CD13, CD29, CD44, CD49f, Thy-1, CD90, CD104, p75NGFR, β2-microglobulin, CD130, STRO-1	CD34, CD38, CD45, CD133	Adipocytes, Chondrocytes, Osteoblasts, Pancreatic endocrines	[78,79]
Synovial fluid	CD10, CD166, CD44, CD54, CD90, CD105, CD147, D7-FIB, STRO-1	CD31, CD34, CD45, CD106, CD117, CD166, VEGFR2, Flk-1, CXCR4, BMPR-1A, NGFR	Adipocytes, Chondrocytes, Osteoblasts	[80,81]
Synovial tissues	CD4, CD34, CD45	CD44, CD73, CD90, CD105	Adipocytes, Chondrocytes, Osteoblasts	[10,11,82,83]
Nasal polyp tissues	CD105, CD90, CD73, CD54, CD44	CD34, CD45, CD117, HLA-DR, PDL-1, PDL-2, CTLA-4, CD106, CD146, CD31	Adipocytes, Osteoblasts, Chondrocytes, Neuronal-like cells	[91,92,93,94]
Endometrium	CD73, CD90, CD105, CD146	CD34, CD45	Adipocytes, Chondrocytes, Osteoblasts	[84,85]
Limb bud	CD13, CD29, CD90, CD105, CD106	CD3, CD4, CD14, CD15, CD34, CD45, HLA-DR	Osteoblasts, Adipocytes, Hepatocytes, Neuronal-like cells	[86]
Peripheral blood	CD44, CD90, CD105, HLA-ABC, CD29, CD73, CD90.1, CD106, CD140α	CD45, CD133, CD34, CD19, CD11b, c-kit	Adipocytes, Osteoblasts, Chondrocytes, Neuronal-like cells	[87,88,89,90]

**Table 2:** Effect of mesenchymal stem cells in inflammatory-related disease animal models.

Disease Model (Generation Methods)	Up-Regulation	Down-Regulation	References
Graft-vs-host disease; Depleting endogenous hematopoietic cells by radiation or chemotherapy	Regulatory T cells	Auto-antibodies Inflammatory cytokines T cell proliferation TH1 cells	[97,98,99,100,101]
Type 1 diabetes (T1D); Treatment of streptozotocin	Regulatory T cells Tissue repair TH2 cells	Inflammatory T cells TH1 cells	[102,103,104,105,106]
Pancreatic islet transplantation Treatment of streptozotocin	Islet survival Regulatory T cells	TH1 cytokines T cell responsiveness	[107,108,109]
Experimental autoimmune arthritis For rheumatoid arthritis, collagen-induced arthritis For osteoarthritis, meniscectomy; ovariectomy; treatment of sodium monoiodoacetate	Regulatory T cells IL-10 TH2 cells	Inflammatory cytokines T cell responsiveness	[110,111,112,113,114]
Experimental autoimmune encephalomyelitis (EAE); Induced CNS inflammation by treatment of complete Freund’s adjuvant	TH2	T cell responsiveness CNS infiltration Auto-antibodies TH1/TH17 cells	[115,116,117,118]
Inflammatory bowel disease (IBD); 1, Treatment with dextran sulfate sodium added to drinking water 2, Intrarectal administration of trinitrobenzene sulfonic acid	Anti-inflammatory cytokines Regulatory T cells FasL-mediated T cell apoptosis	Inflammatory T cells Inflammatory cytokines Intestinal CD4^+^ T cell infiltration Growth factor expression T cell responsiveness	[111,119,120,121,122,123]
Systemic lupus erythematosus (SLE); 1, Progeny of a breeding pair consisting of a New Zealand Black mouse and New Zealand White mouse. 2, Mutation in the gene encoding Fas on the MRL strain background	Regulatory T cells Anti-inflammatory cytokines	Anti-DNA antibodies T cell frequency TH17 cells Plasma cells Inflammatory cytokines	[124,125,126,127]
Chronic rhinosinusitis with nasal polyps (CRSwNP); For eosinophilic CRSwNP, Ovalbumin and *Staphylococcus aureus* enterotoxin B For non-eosinophilic CRSwNP, Lipopolysaccharide	Regulatory T cells IL-10	CD4^+^ and CD8^+^ T cell proliferation IL-2, TNF-α, IFN-γ	[49,128,129,130,131,132]

**Table 3:** Effect of mesenchymal stem cells in fibrosis-related disease animal models.

Disease Model	Route of Delivery	Therapeutic Effect	References
**Lung**			
Bronchopulmoary dysplasia			
Hyperoxia neonatal lung injury	Intravenous, Intratracheal, intraperitoneal	Protection of alveoli, Reduce and decrease inflammation, pulmonary injury, hypertension and fibrosis Vascular growth, Increase survival	[166,167,168,169]
Acute respiratory distress syndrome			
Bacterial pneumonia	Intravenous	Improve oxygenation (PaO_2_/FiO_2_^w^) Decrease pulmonary edema	[162]
LPS-induced inflammation	Intravenous	Reduce histopathological changes, Increased survival, Protection of alveoli, Lung mechanics improve	[170]
Chronic lower respiratory disease			
Cigarette smoke exposure	Intratracheal /Intravenous	Decrease tracheal responsiveness, inflammatory cytokines, and inflammatory cell infiltration	[163]
LPS, cigarette smoke, and 17% oxygen exposure	Intratracheal	Decrease in inflammatory cytokines, Increase in ECM production	[171]
Cycstic fibrosis			
Naphthalene-induced lung injury	Intravenous	Little to no level of CFTR dependent chloride secretion	[164]
Idiopathic pulmonary fibrosis			
Bleomycin-induced lung injury	Intratracheal	Decrease fibrosis and airway inflammation	[165]
**Liver**			
Chronic hepatitis B	Intravenous	Improvement of liver function and MELD score Reduce ascites	[172]
Primary biliary cirrhosis	Intravenous	Decrease in serum ALP and γ-GGT	[173]
Hepatitis C virus cirrhosis	Intravenous infusion, Peripheral vein	Improvement in liver function; Frequency of encephalopathy, jaundice, ascites, bleeding tendency, and lower limb edema	[174,175]
Hepatitis B virus cirrhosis	Hepatic artery	Improvement in liver function	[176]
**Pancreas**			
Dibutyltin dichloride	Penile vein, Jugular vein	Immunomodulatory effect Inhibition of activation of pancreatic satellite cells Anti-apoptotic effect	[177,178,179]
**Heart**			
Ischemic heart failure	Intramyocardial	Reduction of infarct scar, inflammation, vascular permeability, fibrosis in scarred tissues Improve LVEF and endothelial function Increase cardiac function, survival and angiogenesis	[180,181,182,183,184,185]
